# A Modfied Nuss Procedure for Recurrent Pectus Excavatum of Adults

**DOI:** 10.3389/fsurg.2021.814837

**Published:** 2022-01-26

**Authors:** Lei Wang, Rui Bi, Xiao Xie, Haibo Xiao, Fengqing Hu, Lianyong Jiang

**Affiliations:** Department of Cardiothoracic Surgery, Xinhua Hospital Affiliated to Shanghai Jiao Tong University School of Medicine, Shanghai, China

**Keywords:** pectus excavatum (PE), recurrent pectus excavatum, minimally invasive (MI), adult, modified Nuss procedure, Nuss

## Abstract

**Background:**

Limited data exist for adults with recurrent pectus excavatum (PE) treated with minimally invasive surgical repair.

**Methods:**

Between July 2008 and December 2020, forty-two adult patients with recurrent PE underwent a modified Nuss procedure with a newly designed bar in our center. A small vertical subxiphoid incision was used to separate severe adhesions when necessary. Multiple steel wires were sutured, and the rib space was narrowed to firmly fix the bar. The primary end point was Haller index change after operation. The secondary end points included length of stay after operation, short-term and long-term complications.

**Results:**

The mean patient age was 22.02 ± 3.49 years. The mean Haller index was 4.59 ± 1.09. A subxiphoid incision was performed in 12 patients. Thirty-nine patients had one bar placed, and 3 patients required two bars. Sixteen patients had 3 or more wires fixation, and 4 patients needed to have their intercostal space narrowed. There was no perioperative death, and the mean hospitalization was 5.57 ± 2.47 days. The Haller index reduced to 3.03 ± 0.41 after the operation (t = 11.85, *p* < 0.001). During the follow-up, there were 3 patients who developed non-infective wound effusion; bar rotations occurred in 3 patients. Twenty patients had the bar removed, post-bar removal Haller index was significantly reduced compared to the preoperative Haller index (2.89 ± 0.37 vs. 4.72 ± 1.05, *t* = 8.96, *p* < 0.001).

**Conclusions:**

The modified Nuss procedure with a new titanium alloy bar can achieve good results for adult patients with recurrent PE.

## Introduction

Pectus excavatum (PE) is the most common congenital chest wall anomaly and is characterized by a depression in the anterior chest wall and sternum. Surgical correction remains the definitive management of PE, and multiple techniques for repair have been described, such as the Ravitch procedure, Nuss procedure and their modifications (1–5). Regardless of the kind of method we choose, there is always a certain probability of recurrence, which is ~2–10% (6–8). Multiple factors have been thought to be responsible for recurrence after PE repair. Overly extensive or minimal dissection may lead to recurrence after the Ravitch procedure (9), and failure of the Nuss procedure may be due to a displacement of the steel bar or a premature removal of the pectus bar (10).

Some recurrent PE cases require reoperation because of heart compression or symptoms such as chest pain. For children and adolescents, minimally invasive surgery for recurrent PE is always effective because of the pliable chest walls in these patients (11), but for adult patients with recurrent PE, the effectiveness of minimally invasive surgery is controversial due to the rigidity of the chest wall, especially for the Ravtich procedure (12, 13). We modified the Nuss procedure with a newly designed bar, in this study, we examine our institutional experience with adult recurrent PE repair and describe the efficacy of a minimally invasive approach.

## Patients and Methods

The study was approved by the ethics committee of Xinhua Hospital Affiliated to Medical College of Shanghai Jiaotong University (XHEC-D-2021-109), and a retrospective review of all patients undergoing repair of recurrent PE in our hospital between July 2008 and December 2020 was performed. Inclusion criteria: (1) The patient was diagnosed as recurrent PE; (2) The patient was ≥18 years old. Exclusion criteria: (1) The second operation for recurrent PE was the classic Nuss procedure or the Ravitch procedure; (2) The patient had incomplete data records.

The patient demographics, symptom before operation, time and type of previous repair, preoperative imaging and testing, information regarding the operation (number of bars, number of wires, narrow intercostal space or not, need subxiphoid incision or not, operative time, etc.), length of stay after the operation, short-term complications (pneumothorax, pleural effusion, pulmonary infection, atelectasis, wound drainage, incisional infection) and long-term complications (bar displacement/rotation; pectus excavatum recurrence, etc.) were evaluated. The primary end point was Haller index change after operation. The secondary end points included length of stay after operation, short-term and long-term complications.

### New Bar Configuration and Accessories

**Figure 1:**
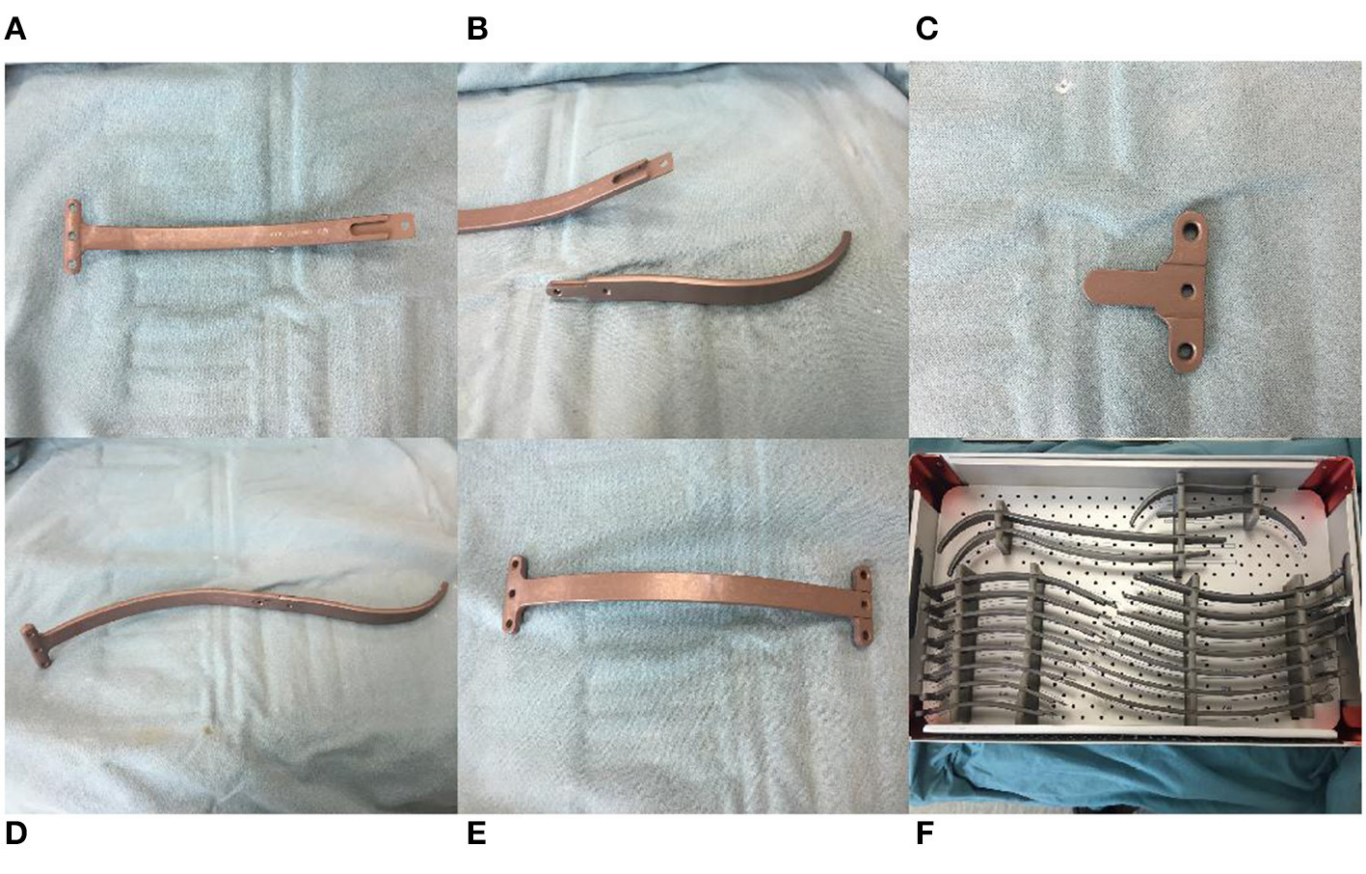
Bar configuration and accessories. **(A)** One end of the titanium alloy bar was fused with a bar stabilizer. **(B)** Introducer of bar. **(C)** Bar stabilizer. **(D)** Bar connect with introducer. **(E)** Bar connect with stabilizer. **(F)** Different sizes of bars.

The new bar (Jiangxi Xingye Medical Devices, Shanghai, China, patent number,CN201481536U) was curved according to the normal structure of the human anterior chest wall, one end of the steel bar was fused with a bar stabilizer, and the other end was designed to connect with the introducer or stabilizer. The bars were divided into large and small sizes according to the different lengths and widths. The bars have 17 different specifications varying from 120 to 280 mm, which are distinguished by the different lengths. Each specification has a difference of 10 mm. The small bars (120–190 mm) are 11 mm wide and 4 mm thick and are often used for children. The large bars (200–280 mm) are 15 mm wide and 4 mm thick and are often used for older adolescents or adults. Compared with the Nuss steel bar, the new bar is shorter. The bilateral stabilizers were fixed at the anterior chest wall rather than the lateral chest wall, the bars had multiple specifications and needed no intraoperative orthopedics, and we could choose different types of bars according to the length of the patient's thorax. Considering that softer steel bars can bear less pressure, which may lead to operation failure, we produced bars thicker than the Nuss steel bars to make the bars harder (4 vs. 2 mm). For the same reason, we used titanium alloy (hardness >260 HV10) instead of steel (hardness >210 HV10) to produce bars in recent years because we have observed that some steel bars were slightly deformed after removal in the early stage (Figure 1).

### Operative Technique

**Figure 2:**
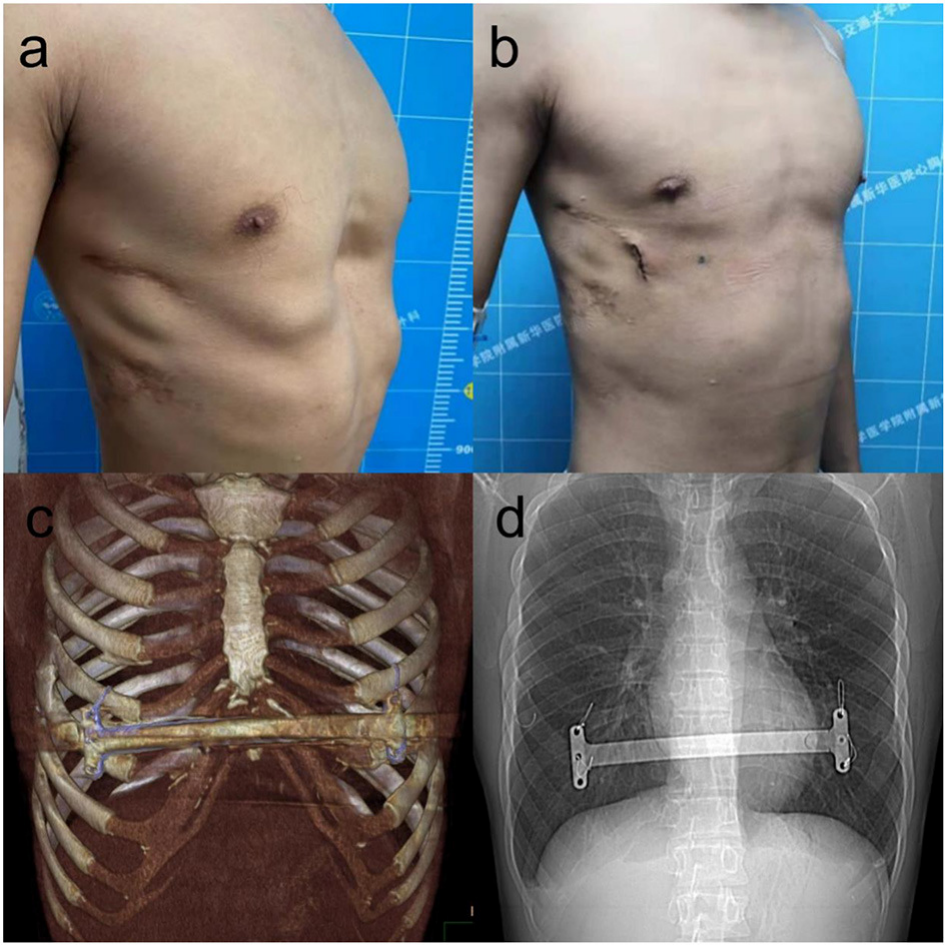
The appearance before **(a)** and after **(b)** reoperation of a 22-year-old patient with recurrent pectus excavatum after Nuss procedure. The bar was placed at anterior chest wall **(c)**, four steel wires fixed bilateral stabilizer of the bar to ensure the stability of the bar **(d)**.

The modified Nuss procedure was first used in 2008, and there have been many improvements in this technique with the accumulation of experience. The patient was placed in the supine position using general anesthesia and orotracheal intubation. The lowest point of the depression, the highest point of chest wall on both sides, and the surgical incision bilaterally were marked, the vertical incisions were always 2 to 3 cm long near the middle axillary line, and an intercostal nerve block was performed around the incision. After routine disinfection and towel laying, an appropriate bar was selected according to the distance between the anterior axillary lines on both sides. Incisions were made bilaterally along the marked line. The subcutaneous tissue around the incisions was dissected to make a pocket so that the stabilizers could be placed, and then the subcutaneous tissue was separated upward to the mark of the highest point of the chest wall on both sides. A 5-mm-diameter thoracoscope was inserted into the right thoracic cavity 1 or 2 intercostal spaces below the incision on the middle axillary line through a trocar to guide and monitor the procedure. In recent years, to further reduce the injury and aesthetic considerations, we placed the trocar into the right surgical incision. After the right lung was collapsed, steel wires or sutures were sutured to fix the bar. In the early stage and because of the lack of experience, the bar was fixed with sutures. In recent years, the bar is fixed with 6 mm steel wires, and we suture 1–2 steel wires according to the hardness of the chest wall and the surgeon's experience. After the right steel wires were placed, sutures of 1–2 steel wires or sutures were placed on the left side in the same way for standby. After the bar was tied to the end of the introducer, the introducer was inserted into the right thoracic cavity from the highest point of the right chest wall, the posterior sternal tissue was separated under the guidance of thoracoscopy, the introducer was pulled out from the highest point of the left chest wall, the introducer was removed, and then a stabilizer was placed on the left side of the bar to support and fix the bar. Finally, the stabilizers were tied to the ribs with wires or sutures bilaterally to avoid bar rotation (Figure 2).

**Figure 3:**
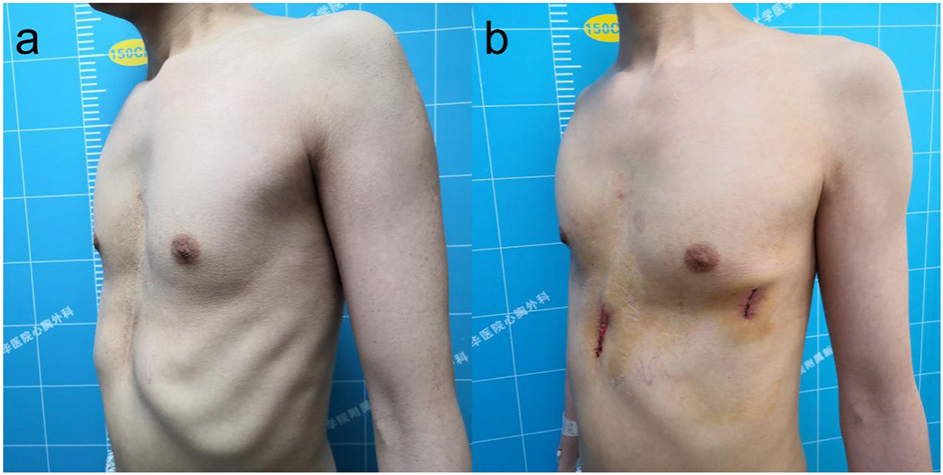
The appearance before **(a)** and after **(b)** reoperation of a 33-year-old patient with recurrent pectus excavatum after Ravitch procedure.

**Figure 4:**
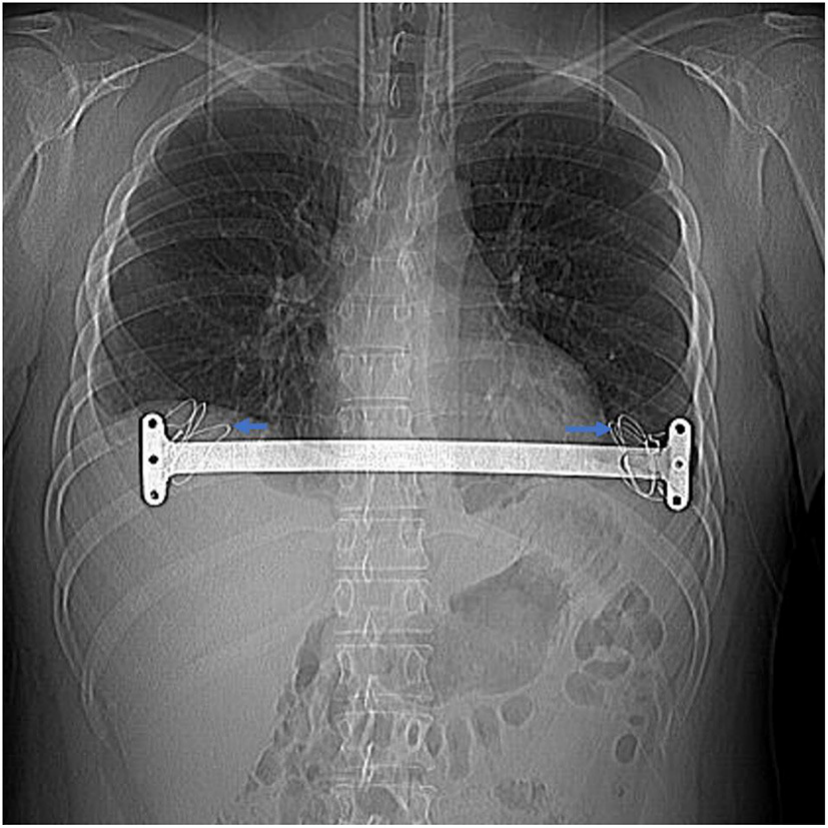
Bilateral intercostal space was narrowed with steel wires when intercostal muscle tear (shown by the arrow) in a 24-year-old patient with recurrent pectus.

If the adhesion of the thoracic cavity or retrosternal space affects the placement of the bar, an electric hook or an ultrasonic scalpel was used to separate the adhesion until a tunnel was established, and then we placed the bar. If the retrosternal adhesion was dense and difficult to separate, to avoid damage to the heart, a small vertical subxiphoid incision was made to separate the retrosternal adhesions with the surgeon's fingers and an electric knife, and the introducer of the bar could be placed through the retrosternal space guided by the surgeon's finger (Figure 3). If tears in the intercostal muscles resulted in sinking and displacement of the bar during operation, we used steel wires to narrow the intercostal space below the penetration and/or exit points of the bar (Figure 4) to provide better support for the bar. Meanwhile, considering that the bar may displace easier in this type of situation, it is necessary to fix as many steel wires as possible to avoid bar displacement. Finally, any air in the pleural cavity was evacuated by producing a large tidal volume and by applying suction through a chest tube placed into a trocar hole. A self-controlling analgesia infusion pump was used to alleviate the postoperative pain for 2 or 3 days. All patients received chest roentgenograms on the day of the operation, and complications such as pneumothorax and pleural effusion. were recorded. All patients were followed up for 3 months after the operation to observe wound healing and bar displacement *via* chest CT. Patients were advised to have the bar removed 3 years after the operation.

### Statistical Analysis

SPSS 18.0 statistical software was used for the data analysis. Continuous variables with a normal distribution are expressed as the mean ± standard deviation. The count data are expressed as the number of cases (n) and percentage (%). The comparisons among normally distributed continuous variables were conducted *via t*-tests, and *p* < 0.05 was considered to be statistically significant.

## Results

A total of 46 adult patients with recurrent pectus excavatum were included in the study, and 4 patients were excluded: one patient was excluded due to being treated with the classic Nuss procedure, one patient also had pectus carinatum that required a simultaneous operation, and the other two patients had incomplete records (preoperative CT data were not available). A total of 42 patients with recurrent pectus excavatum were treated with our modified Nuss procedure, including 36 male patients. The mean patient age was 22.02 ± 3.49 years (range 18–30 years), with a mean Haller index of 4.59 ± 1.09 (range, 2.95–7.21). The most common presenting symptoms were shortness of breath (57.14%) and chest pain (19.05%). The main comorbidities were complete or incomplete right bundle branch block (23.81%), tricuspid regurgitation (14.29%) and scoliosis (11.90%). The demographic data, symptoms, comorbidities and operation history of the patients are shown in Table 1.

**Table 1:** Demographic variables and preoperative characteristics of the study population.

**Variables**	**Patients (*n* = 42)**
Age, years (mean)	22.02 ± 3.49 (18–30)
Sex, n (%)	
Male	36 (85.71%)
Female	6 (14.29%)
Preoperative symptoms, *n* (%)	
No symptom	12 (28.57%)
Shortness of breath	24 (57.14%)
Chest pain	8 (19.05%)
Asthma/asthma-like symptoms	2 (4.76%)
Previous operation, *n* (%)	
Ravitch	21 (50%)
Nuss	19 (45.24%)
Ravitch and Nuss	2 (4.76%)
Open heart surgery	1 (2.38%)
Other chest surgery	2 (4.76%)
Comorbidity, *n* (%)	
Complete or incomplete right bundle branch block	10 (23.81%)
Tricuspid regurgitation	6 (14.29%)
Scoliosis	5 (11.90%)
Mitral regurgitation	2 (4.76%)
Pulmonary bullae	2 (4.76%)
Time after primary procedure (range), years	8.18 ± 6.50 (0.5–25)
Preoperative Haller index (range)	4.59 ± 1.09 (2.95–7.21)

All surgical procedures were completed without major intraoperative complications. Thirty patients completed the modified Nuss procedure without a subxiphoid incision, and 12 cases of recurrent PE required a subxiphoid incision. One patient underwent cardiac surgery, and the Ravitch procedure was converted to a sternotomy because of dense adhesions between the sternum and right ventricle. We separated the dense adhesions under a cardiopulmonary bypass to avoid fatal hemorrhage. In one patient after the Ravitch procedure, the depressed anterior chest wall could not be elevated adequately because of the rigid sternum after we placed the bar, so we had to prolong the subxiphoid incision and perform a transverse sternal osteotomy. The deformity was finally completely corrected.

In this study, 39 patients had only one bar placed, and 3 (7.14%) patients required two bars because of the wide range of the depressions. Twenty-three patients had 2 wire fixations, and 3 or more wire fixations were required in 16 patients. Intercostal muscle tears resulting in a downward displacement of the bar were found in 4 patients after we placed the bar, and the sternum could not be adequately elevated. We narrowed the intercostal space and completely corrected the pectus excavatum in these patients. The intraoperative information, complications and postoperative hospital stay are shown in Table 2.

**Table 2:** Intraoperative variables and data from the hospital stay.

**Variables**	**Patients (*n* = 42)**
Operative time, min (mean)	90.86 (35–195)
Bars placed, *n* (%)	
Single	39 (92.86%)
Double	3 (7.14%)
Wires fixation, *n* (%)	
0	2 (4.76%)
1	1 (2.38%)
2	23 (54.76%)
3	6 (14.29%)
4	10 (23.81%)
Need a subxiphoid incision, *n* (%)	12 (28.57%)
Transverse sternal osteotomy, *n* (%)	1 (2.38%)
Remove bars of initial operation, *n* (%)	5 (11.90%)
Narrow intercostal space, *n* (%)^*^	4 (9.52%)
Mortality, *n* (%)	
Intraoperative death	0 (0%)
30-day mortality	0 (0%)
Length of hospital stay after operation, days (mean)	5.57 (3–15)

* *, wires narrow intercostal space was not include in wires fixation*.

The short-term postoperative complications mainly included pleural effusion and pneumothorax, but most patients had only a small amount of pleural effusion or pneumothorax and needed no surgical intervention. Only 2 patients needed to receive drainage tubes. All patients received a CT examination in the third month after the operation, and the Haller index (postoperative Haller index) was measured in all of the patients. The results showed that the Haller index of the patients had decreased significantly after the operation (preoperative Haller index vs. postoperative Haller index, 4.59 ± 1.09 vs. 3.03 ± 0.41, *t* = 11.85, *p* < 0.001).

All patients were followed up for 64.31 ± 35.87 months (range 10 to 121 months). During the follow-up, there were 3 patients that had no-ninfectious wound effusion that were cured after debridement. Bar rotation occurred in 3 patients, but the rotation angle was small, there was no depression observed in the anterior chest wall, and none of the patients needed a second operation. One patient needed a second bar insertion, who was a female patient with extensive pectus excavatum with a slight depression of the upper chest wall and an extensive depression of the lower chest wall. We only placed one bar in the operation, but the patient was not satisfied with the slight depression of the upper chest wall, so we placed another bar for her. One patient had the bar removed 29 months after the operation because of severe pain after exercise. The Haller index was 2.66 after removing the bar, and the pectus excavatum did not recur during the follow-up. As of December 2020, 20 of the patients had the bar removed, which was an average of 43.5 months after bar placement, and the post-bar removal Haller index was significantly reduced compared to the preoperative Haller index (Preoperative Haller index vs. post-bar removal Haller index,4.72 ± 1.05 vs. 2.89 ± 0.37, *t* = 8.96, *p* < 0.001). The early and late complications and Haller index are shown in Table 3.

**Table 3:** Early and late postoperative complications.

**Variables, *n* (%)**	**Patients (*n* = 42)**
Early complications	
Bleeding requiring transfusion or reoperation	0 (0%)
Pleural effusion	20 (47.62%)
Pneumothorax	15 (35.71%)
Pneumonia	5 (11.90%)
Atelectasis	8 (19.05%)
Place drainage tube after operation	2 (4.76%)
Late complications	
Wound effusion	3 (7.14%)
Wound infectious	0 (0%)
Bar displacement	3 (7.14%)
Required 2nd bar insertion	1 (2.38%)
Required early bar removal	1 (2.38%)
Postoperative Haller index	3.03 (2.18–3.82)
Late Haller index	2.89 (2.14–3.61)
Bar removal after bar placement (month)	43.50 (29–84)

## Comment

Ravitch described the classical operative approach to the management of pectus excavatum in 1949. The procedure involves the resection of the deformed costal cartilage, a xiphoid division from the sternum, and transverse sternal osteotomy to displace the sternum anteriorly (1). Although the method has achieved good results, it has disadvantages, including a long operating duration, a long hospitalization, a large amount of blood loss, and the scarring of the anterior chest wall (14, 15). Donald Nuss introduced a minimally invasive approach to repair pectus excavatum in 1998 (2). This technique involves correction of the sternal defect with a convex steel bar inserted through small bilateral thoracic incisions under thoracoscopic visualization. The Nuss procedures have been widely used as standard minimally invasive procedures to treat PE because they are less invasive and have more cosmetic effects than the open methods. The success of the Nuss procedure relies on the chest wall pliability, which may have been diminished due to the age of patients (16). It is more difficult to elevate the sternum in adult patients than in child patients with pectus excavatum, and this is especially true in adult patients who have had prior chest wall reconstructive surgery (17). Moreover, previous operations may cause thoracic and retrosternal adhesions, which bring difficulties during the second operation. How to treat the thoracic and retrosternal adhesions caused by the first operation and how to achieve adequate sternal elevation are the keys to ensuring the success of the second minimally invasive repair of recurrent PE in adults.

The Nuss procedure needs to establish a tunnel in order to insert the bar, and the process may be affected by the thoracic and retrosternal adhesions secondary to the previous operation. The intrathoracic dense adhesions between the sternum and mediastinal structures increase both the difficulty and the potential risks of reoperative procedures (18).When the bar passes through the mediastinum, it can produce a fatal cardiac injury (3, 12, 19).Two patients in our center had heart rupture during the separation of sternal adhesions (not included in this study because they were younger than 18 years old), one patient had extremely severe pectus excavatum after the Ravitch operation, the patient's heart was severely compressed and deformed, and the right atrium ruptured during separation with the introducer. The other patient had undergone congenital heart disease surgery, the pericardium was partially removed, and the right ventricle ruptured during the separation of the adhesion between the right ventricle and the sternum. How to avoid fatal cardiac injury in a second operation is a challenge for every surgeon, and some surgeons choose to suspend the sternum or chose an open surgery to avoid the risks (12, 13). In our modified Nuss operation, to avoid the risk, a small incision was made under the xiphoid, the surgeon's fingers and electric knife were used to separate the retrosternal adhesions before we introduced the bar through the retrosternal space, and the surgeon's fingers could be used to guide the bar when the bar passed into the mediastinum through the incision. In addition, we can transform a median sternal incision in a timely manner when necessary, and previous reports also advocate using a subxiphoid incision to decrease heart injuries (3, 20, 21). In this study, 12 patients had posterior sternal adhesions separated under a subxiphoid incision, 2 patients underwent the Nuss procedure, and both cases had severe pectus excavatum compressing the right ventricle. It was difficult to observe the posterior sternum tissue under thoracoscopy, so we made a subxiphoid incision to separate the retrosternal space and to guide the bar to insert it through the posterior sternal space with a finger. The remaining 10 cases were patients who previously had the Ravitch operation. It was difficult to separate the dense posterior sternal adhesions by electric hook or ultrasonic scalpel under thoracoscopy, so we had to adopt a subxiphoid incision. It is simple and safe to separate the retrosternal space by this method. We feel that this modification diminishes the chance of serious injury to the heart as the bar passes through the mediastinum, and no massive hemorrhage occurred in this group.

The principle of the Nuss procedure is to elevate the depressed anterior chest wall to a normal position with one or more bars. The main support point is the two ribs and intercostal muscle under the bar, similar to the piers of a bridge. One bar is enough for most patients with soft chest walls, but it may not be enough for patients with rigid chest walls, especially in adult patients with recurrent pectus excavatum. Therefore, it is often necessary to place two or more bars in different intercostal spaces to increase the support strength (2, 11, 22).Compared with the Nuss steel bar, the bar we designed was shorter, and there were two stabilizers on both sides of the bar. The bar could be placed on the upper and lower ribs of the intercostal spaces, so there were four ribs supporting the bar at the same time. In addition, considering that a soft bar is easily deformed under pressure, which may lead to operation failure, the bar we designed does not need to be shaped during operation, so we manufactured the bar to be thicker, which makes the bar harder and can allow it to withstand a greater pressure. Although we observed that some steel bars were slightly deformed when they were removed, we manufactured bars with titanium alloy instead of steel in recent years because the hardness of titanium alloy is more than that of steel. In this group of recurrent adult pectus excavatum, for most patients, one bar was enough to adequately elevate the sternum, only 3 patients (7.14%) with wide range pectus excavatum deformities needed two bars, and no patients needed 3 bars.

In addition to the strength of the support, the stability of the bar is also very important, because the recurrence of pectus excavatum after the Nuss procedure is mostly due to rotation or displacement of the bar (23–25). In this group, 5 patients who underwent the initial Nuss procedure experienced recurrence because of bar displacement. Considering this happens type of bar rotation is mainly due to insufficient fixation, we improved the surgical technique by using multiple steel wires to fix the bar to ribs, with at least 2 wires. For patients with pectus excavatum who have rigid chest walls, we fixed 3 or 4 steel wires to ensure the stability of the bar. In this group, bars were fixed with 3 or more wires in 16 patients. During the follow-up, bar rotation occurred in 3 patients, one patient had bar rotation that occurred in the early stage, and we fixed the bar with thread. In the other 2 patients, the bars were fixed with 2 wires, and no bar displacement occurred in 16 patients with fixed bars with 3 or more wires. We think 2 wires sometimes may not be enough, and multiple wire fixation is necessary for adult patients with recurrent pectus excavatum to avoid bar displacement.

Moreover, the protection of intercostal muscles is also very important (26). The tearing of intercostal muscles may also lead to bar displacement. This phenomenon rarely occurs in children, but it may occur in some adult patients due to the great pressure that the bar needs to bear during surgery, especially in patients with a wide intercostal space and weak intercostal muscles, which will lead to the downward displacement of the bar. Although the bar does not rotate or turn over, the bar cannot provide enough upward support strength to the sternum, and this may lead to the failure of the operation. In this study, intercostal muscle tears occurred in 4 patients, so we had to suture steel wires under the bar to narrow the rib space. Meanwhile, multiple wire fixation was also necessary to stabilize the bar in these patients.

Considering that the removal of the bar too soon may lead to the recurrence of pectus excavatum, we generally recommend patients to have the bar removed 3 years after surgery, however, compared with the patients with a primary operation, patients with recurrent PE always have greater psychological pressure. Many of these patients are worried about the recurrence of pectus excavatum after having the bar removed, so they retain the bar longer than 3 years. In this group, 20 patients have so far had the bar removed, which is an average of 43.5 months after surgery. The Haller index improved significantly, from average 4.72 to 2.89 when we removed the bar, and there was no recurrences during follow up after we removed the bars.

In summary, it is very safe to separate the dense adhesion behind the sternum through a subxiphoid incision after the primary operation. The new bar can withstand a greater pressure, we can reduce the number of bars used in the operation, and multiple fixations of the bar and narrowing of the intercostal space can prevent displacement of the bar. The modified Nuss procedure with the new bar can achieve good results for adult patients with recurrent pectus excavatum after the Nuss procedure or the Ravitch procedure. However, there are still some limitations in this study, it is a retrospective single-center study with a small number of cases, a multiple-center prospective study with large number of cases will be needed to further drawn our conclusion.

## Data Availability Statement

The original contributions presented in the study are included in the article/supplementary material, further inquiries can be directed to the corresponding author/s.

## Ethics Statement

The studies involving human participants were reviewed and approved by the Ethics Committee of Xinhua Hospital Affiliated to Medical College of Shanghai Jiaotong University. The patients/participants provided their written informed consent to participate in this study.

## Author Contributions

All authors listed have made a substantial, direct, and intellectual contribution to the work and approved it for publication.

## Conflict of Interest

The authors declare that the research was conducted in the absence of any commercial or financial relationships that could be construed as a potential conflict of interest.

## Publisher's Note

All claims expressed in this article are solely those of the authors and do not necessarily represent those of their affiliated organizations, or those of the publisher, the editors and the reviewers. Any product that may be evaluated in this article, or claim that may be made by its manufacturer, is not guaranteed or endorsed by the publisher.
